# Understanding the Sorghum–*Colletotrichum sublineola* Interactions for Enhanced Host Resistance

**DOI:** 10.3389/fpls.2021.641969

**Published:** 2021-04-20

**Authors:** Kibrom B. Abreha, Rodomiro Ortiz, Anders S. Carlsson, Mulatu Geleta

**Affiliations:** Department of Plant Breeding, Swedish University of Agricultural Sciences, Alnarp, Sweden

**Keywords:** sorghum, anthracnose, quantitative trait loci, *R*-genes, germplasm, host-plant resistance, *Colletotrichum sublineola*

## Abstract

Improving sorghum resistance is a sustainable method to reduce yield losses due to anthracnose, a devastating disease caused by *Colletotrichum sublineola*. Elucidating the molecular mechanisms of sorghum–*C. sublineola* interactions would help identify biomarkers for rapid and efficient identification of novel sources for host-plant resistance improvement, understanding the pathogen virulence, and facilitating resistance breeding. Despite concerted efforts to identify resistance sources, the knowledge about sorghum–anthracnose interactions remains scanty. Hence, in this review, we presented an overview of the current knowledge on the mechanisms of sorghum-*C. sublineola* molecular interactions, sources of resistance for sorghum breeding, quantitative trait loci (QTL), and major (*R*-) resistance gene sequences as well as defense-related genes associated with anthracnose resistance. We summarized current knowledge about *C. sublineola* populations and its virulence. Illustration of the sorghum-*C. sublineola* interaction model based on the current understanding is also provided. We highlighted the importance of genomic resources of both organisms for integrated omics research to unravel the key molecular components underpinning compatible and incompatible sorghum–anthracnose interactions. Furthermore, sorghum-breeding strategy employing rapid sorghum germplasm screening, systems biology, and molecular tools is presented.

## Introduction

**Figure 1:**
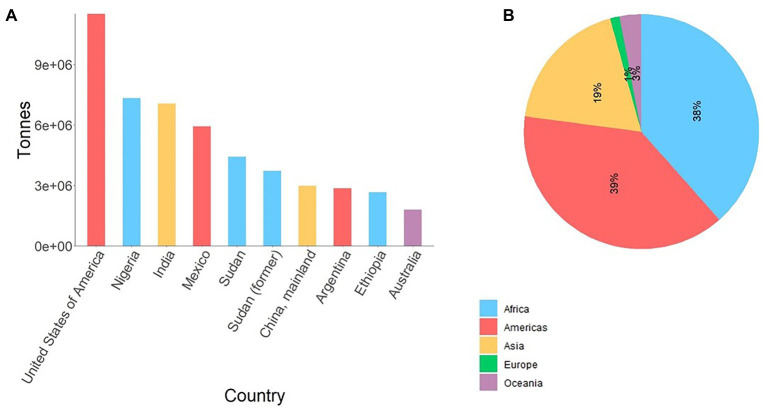
**(A)** Average production of sorghum in top 10 sorghum producing countries from 1994 to 2018, and **(B)** corresponding production share by region. Data source (FAOSTAT, 2020). Sudan (former) reflects average sorghum production in Sudan and South Sudan up to 2011. For Sudan, the data represent average production from 2012 to 2018.

Sorghum [*Sorghum bicolor* (L.) Moench] – a diploid photosynthesis efficient C_4_ crop – is one of the most important cereals serving as a staple food for over 500 million people globally, used as animal feed, and is increasingly important source of biomass for cellulosic ethanol production. Due to its rich genetic diversity and adaptability to adverse conditions such as drought, nowadays sorghum is annually cultivated on over 42 million ha across six continents (FAOSTAT, 2020). In spite of its diverse use and resilience, sorghum is predominantly produced by subsistence farmers in the developing world (Figure 1) significantly contributing to food security. However, biotic and abiotic stresses are causing significant yield losses across all its growing areas.

Diseases, such as stalk rot, downy mildew, grain mold, rust, head smut, leaf blight, and anthracnose are constraining global sorghum production (Wang et al., 2006; Little et al., 2012; Tesso et al., 2012; Das and Rajendrakumar, 2016; Mengistu et al., 2018). Because of its wide distribution and ability to infect all above ground parts of the plant, anthracnose caused by the destructive fungal pathogen *Colletotrichum sublineola* is one the most important diseases of sorghum. Since it was first reported in Togo in 1902 and later in United States in 1912 (Crouch and Tomaso-Peterson, 2012), the pathogen has spread to almost everywhere where sorghum is grown. It is causing significant yield losses annually, especially in the tropical and subtropical regions where there are favorable climatic conditions for disease development (Erpelding, 2008). Yield losses of up to 67% due to anthracnose are recorded in susceptible sorghum cultivars (Mengistu et al., 2018) but, without efficient disease management practices, the pathogen can devastate the whole crop.

Agronomic practices alone are not effective to reduce infections and yield losses due to anthracnose. Although fungicide application is an effective method to control the disease and reduce yield losses (Acharya et al., 2019), it is not economically and practically feasible for small-scale farmers and is not environmentally friendly. Growing sorghum cultivars resistant to anthracnose is considered the most efficient and is a core in integrated strategy for anthracnose management. To this end, improving anthracnose resistance has been an utmost priority in sorghum breeding programs. Fortunately, there is a high genetic diversity and wide anthracnose resistance variation in sorghum landraces (Motlhaodi et al., 2017; Afolayan et al., 2019; Cuevas and Prom, 2020; Mengistu et al., 2020), which can be explored and used in its breeding program for improving resistance against the disease.

Traditional breeding is a relatively slow process and not sufficient to tap full potential of crop genetic resources. The use of DNA markers may increase the pace and efficiency of plant breeding (Zheng et al., 2011; Upadhyaya et al., 2013; Kage et al., 2016; Ordonio et al., 2016). Accordingly, identifying naturally occurring major disease resistance genes (*R*-genes) in wild relatives and landraces of a crop and introducing these genes into elite materials using molecular tools are the most widely adopted strategy to develop disease-resistant cultivars of several crops. The sorghum genome contains hundreds of putative *R*-gene sequences (Mace et al., 2014; McCormick et al., 2018), some of which are localized within the anthracnose resistance associated quantitative trait loci (QTL) regions that were identified in several landraces (Upadhyaya et al., 2013; Felderhoff et al., 2016; Patil et al., 2017; Xu, 2019). These findings suggest the presence of rich sorghum genetic resource that can be used for enhancing sorghum anthracnose resistance. However, none of these *R*-genes have been functionally validated for resistance against *C. sublineola* and introduced into sorghum elite cultivars so far.

Deep understanding of the sorghum–*C. sublineola* interactions is needed in order to rapidly identify resistance sources in the diverse landraces, isolation, and characterization of the *R*-genes (Xu, 2019), and elucidate the virulence of the pathogen (Buiate et al., 2017). In line with this, improving our knowledge on molecular mechanisms of the interaction process can facilitate resistance cultivar development and design an effective disease management strategy against anthracnose. So far, little is known about the underlying molecular events underpinning the sorghum–*C. sublineola* interactions and the existing findings remain scattered. In this review, we present an overview of the current knowledge on molecular-level sorghum–*C. sublineola* interactions. This article summarizes the *R*-genes and QTL associated with the resistance as well other anthracnose defense-related genes in sorghum. It also provides a summary of current knowledge related to anthracnose population structure and virulence, which would be crucial for the improvement of the crop’s resistance against the pathogen. Further, it highlights the potential use of current advances in omics techniques to enhance the understanding of the interaction process and suggests future directions to accelerate the breeding of resistant sorghum cultivars.

## Sorghum Genetic Resources For Enhancing Anthracnose Resistance

Crop germplasm contains genetic makeup of a specific species. Phenotypic and genotypic diversity in a crop germplasm is a foundation for improving its agronomic traits, such as biotic and abiotic stress tolerance, yield, and nutritional quality. A large sorghum germplasm collection that has a paramount importance in sorghum breeding is available (Upadhyaya et al., 2016). Globally, over 236,000 sorghum accessions are maintained in several gene banks (Upadhyaya et al., 2016), of which more than 44,000 sorghum accessions originating from 114 countries, are conserved in the National Plant Germplasm System (NPGS) in United States (Cuevas et al., 2017). Although there could be some overlaps with the NPGS germplasm collection, the International Crops Research Institute for the Semi-Arid Tropics (ICRISAT) in India also maintains 39,923 sorghum accessions (Upadhyaya et al., 2017). Sorghum germplasm collections also exist in several countries that are recognized as its center of origin and/or diversity, such as Ethiopia, Eritrea, and Sudan (Girma et al., 2019; Cuevas and Prom, 2020; Mengistu et al., 2020). However, sorghum wild relatives that have a potential to serve as promising sources of genes for sorghum improvement (Ananda et al., 2020) remain neglected (Upadhyaya et al., 2016). The remarkable phenotypic and genetic diversity within these collections suggest presence of rich sorghum genetic resources. Phenotyping and genotypic characterization of the crop landraces and their wild relatives is a fundamental step for the identification of genotypes that serve as a novel source of resistance in sorghum breeding programs (Upadhyaya et al., 2016).

A diverse sorghum germplasm is crucial genetic resource for breeding programs aimed to improve sorghum anthracnose resistance (Cuevas et al., 2017). Aiming to identify novel sources of anthracnose resistance within the diverse sorghum germplasm, several large-scale mass resistance-screening assays have identified anthracnose resistant genotypes with potential use in sorghum breeding. Thakur et al. (2007) evaluated anthracnose resistance of 15 sorghum lines originating from six countries in 14 anthracnose hotspots in Africa and Asia for 4–7 years, and identified line IS 12467 and IS 6928 that potentially harboring different resistance genes and hence could be integrated into sorghum breeding programs. Anthracnose resistance evaluation of 87 sorghum lines and 63 hybrids against 12 isolates under field conditions shows vertical and horizontal resistance of the host (Buiate et al., 2010). These results indicate the presence of major and minor additive genes in the sorghum germplasm conferring race specific and race nonspecific resistance, respectively. Developing and deploying cultivars with both types of resistance against anthracnose would enhance its efficacy and durability. Anthracnose resistant and susceptible accessions were identified in sorghum germplasm randomly selected from USDA-ARS NPGS (Prom et al., 2012a; Cuevas et al., 2014), Burkina Faso and South Africa (Cuevas et al., 2016), Ethiopia (Mengistu et al., 2019), and China (Xu et al., 2020). Resistance evaluation of sorghum germplasm originating from various geographical regions using several strains of anthracnose, collected from diverse agro-ecologies, across growing seasons would lead to the identification of a highly useful resistance sources for developing sorghum cultivars with wide adaptation. Such studies would diversify potential sources of resistance for sorghum breeding and facilitate efficient utilization of the germplasm. However, despite the efforts by Thakur et al. (2007), there is lack of concerted effort to evaluate accessions across contrasting environments, harboring different *C. sublineola* populations, to identify resistance sources useful for global sorghum breeding. Overall, resistance evaluation studies have a great potential to identify resistance sources that can be directly used in resistant cultivar development through conventional plant breeding or the application of molecular breeding tools, and are highly useful for understanding the inheritance of the anthracnose resistance in sorghum.

## Inheritance of Anthracnose Resistance in Sorghum

Understanding the inheritance of anthracnose resistance in sorghum is a key to identify novel sources, quickly transfer the resistance into elite materials, and enhance durability of introduced resistance to cope up with the diverse and evolving pathogen populations. In line with this, a single dominant gene for resistance against *C. sublineola* was first identified in 1950 (Lebeau et al., 1950). The presence of QTLs associated with the resistance as well as wide variability of the resistance phenotype found in sorghum landraces suggest that anthracnose resistance is a multigenic trait (Cuevas et al., 2014; Ahn et al., 2019; Mengistu et al., 2019; Cuevas and Prom, 2020; Xu et al., 2020). Nevertheless, the anthracnose resistance in sorghum mostly exhibits dominant genetic inheritance even if it also segregates as a recessive trait (Mehta et al., 2005; da Costa et al., 2011).

Using F_3_ population derived from a susceptible (BTx623) and resistant (SC326-6) parents, Boora et al. (1998) reported that the anthracnose resistance is inherited as a single gene recessive trait. Two random amplified polymorphic DNA (RAPD) markers, OPD 16 and OPD 12, were found linked to a recessive resistance allele in sorghum accessions (Panday et al., 2002). A sequence characterized RAPD marker OPJ 011437 is linked to the anthracnose resistance segregating as a recessive in sorghum (Singh et al., 2006). Identification and characterization of these recessive genes conferring resistance would be crucial in order to apply loss-of-function mutation strategies in their dominant homologs to achieve resistance against anthracnose. Although DNA markers found linked to the recessive anthracnose resistance trait in sorghum genotypes are limited, several studies have identified markers associated with the resistance segregating as a dominant trait (Mohan et al., 2010; Burrell et al., 2015; Felderhoff et al., 2016; Cuevas et al., 2017). These markers linked to both forms of resistance trait are crucial resources for marker-assisted selection (MAS) of accessions with novel resistance and their utilization in molecular breeding for developing anthracnose resistant sorghum cultivars. Nevertheless, despite the presence of draft genome sequence of sorghum for over a decade (Paterson et al., 2009), the identified markers have not been validated and utilized in sorghum molecular breeding. This is due to limited efforts to improve the genome annotation, with only few genotypes whole genome-sequenced so far to capture genome-wide sequence polymorphism. Moreover, most of the identified markers are linked to QTLs making it challenging to validate them across environments.

It is worth noting that some sorghum genotypes displaying dominant resistance could also harbor additional resistance genes that show recessive inheritance. Hence, molecular characterization of the variable resistance phenotypes in sorghum would help identify genotypes containing only a dominant resistance gene, or a recessive gene, or both. Classification of the diverse landraces into the three groups, based on levels of disease resistance, is helpful for efficient utilization of the gene pool for sorghum resistance improvement. For instance, since deploying the dominant resistance may increase virulence alleles of the pathogen, augmenting resistance with recessive resistant genes in breeding programs may enhance durability of the anthracnose resistance. However, most sorghum breeding efforts so far have focused on the identification of dominant resistance, mostly conditioned by major resistance (*R*-) genes, because its effect is easier to characterize phenotypically.

## Molecular Aspects Of Sorghum–*C. sublineola* Interactions

Plants are continuously interacting with myriad of microbes and have evolved mechanisms to fend off pathogen attack. The plant response to pathogen infection is generally described by the co-evolutionary zigzag model of plant-microbe interactions (Jones and Dangl, 2006). Describing the complex sorghum-*C. sublineola* interactions within the frame of the zigzag model would help illustrate the current understanding of the pathosystem and elucidate molecular basis of the host resistance and virulence of the pathogen during different phases of the interaction processes.

The *C. sublineola* infection processes on sorghum leaves and disease development have been previously described using cytological and ultrastructural studies (Wharton and Julian, 1996; Wharton et al., 2001), and elegantly reviewed by Crouch and Beirn (2009). *C. sublineola* is a hemibiotrophic pathogen that requires an initial biotrophic phase and, if the infection is successful, transition to a necrotrophic phase happens at ca. 66 h after inoculation (Wharton et al., 2001). Although it remains to be elucidated, the transition could be facilitated by *C. sublineola* plant cell wall (CW) degrading enzymes (CWDEs; Wharton and Julian, 1996; Wharton et al., 2001) to facilitate the infection process*. Sorghum* responds to this initial stages of the *C. sublineola* infection by cell wall apposition forming papillae and accumulation of polyphenolics, phytoalexins, callose, hydrogen peroxide (H_2_O_2_), and hydroxyprolinerich glycoproteins (HRGPs; Basavaraju et al., 2009). Several studies also reported higher expression of several defense related genes encoding pathogenesis-related protein 10 (PR10), chitinase (PR3), and chalcone synthase, and thaumatin-like-protein (TLP; Lo et al., 1999; Li et al., 2013; Ahn et al., 2019). These observed defense responses against pathogen infection are hallmarks of the host basal defense response termed as pathogen/microbe-associated molecular patterns (PAMPs/MAMPs) triggered immunity (PTI) reported in several plants (Jones and Dangl, 2006). PTI is activated when the PAMPS, conserved molecular signatures in the cell wall, are recognized by the pattern recognition receptors (PRRs) on the host cell surface (Jones and Dangl, 2006). The presence of enhanced chitinase (PR3) and β-1,3-glucanase in sorghum plants inoculated with *C. sublineola* (Li et al., 2013; Ahn et al., 2019), for instance, may indicate that chitin and β-glucan in the pathogens’ cell wall serve as PAMPs (Fesel and Zuccaro, 2016) and have been detected by the host receptors and triggered defense response. Biochemical studies characterizing *C. sublineola* cell wall composition and sorghum receptors, which recognize the pathogen and activate host defense response, would provide crucial insights into early molecular events of the interaction process.

The basal defense response against anthracnose appears similar in the resistant and susceptible sorghum genotypes (Wharton et al., 2001). However, the pathogen can successfully penetrate the papillae, invade adjacent host cells, continue colonizing host tissue, and develops a disease in the susceptible genotypes (Wharton and Julian, 1996; Wharton et al., 2001; Basavaraju et al., 2009). The presence of small-secreted proteins, some of which annotated as effectors related to virulence of the pathogen, in the *C. sublineola* genome (Buiate et al., 2017) suggests that the pathogen could secrete plethora of these proteins to manipulate the host defense. Such proteins evading the host response are known as virulence (avr) effectors triggering susceptibility (ETS; Jones and Dangl, 2006). In contrast, hypersensitive response around the infection site due to accumulation of hydrogen peroxide and localized programmed cell death reinforces the PTI defense in the resistant sorghum genotypes (Wharton and Julian, 1996; Basavaraju et al., 2009). The defense response in resistant sorghum genotypes significantly reduces the germination of conidia, formation of appressorium, blocks penetrations, disrupts the pathogen cells, and restricts the pathogen growth at the biotrophic phase (Basavaraju et al., 2009; Tugizimana et al., 2018, 2019). Stronger basal defense response in resistant genotypes could be attributed to earlier accumulation, and higher quantity and diversity of the phytoalexins and expression of defense-related genes (Lo et al., 1999; Basavaraju et al., 2009), probably conditioned by higher efficiency of pathogen detection and signal transduction in the resistant genotypes. In addition, stronger reaction in the resistant genotypes could be related to recognition of the *C. sublineola* secreted proteins, known as avirulence (Avr) effectors, by the host nucleotide binding (NB)-leucine reach repeat (LRR; NB-LRR) encoding genes (Jones and Dangl, 2006). The recognition activates the effector triggered immunity (ETI), characterized by elevated accumulation of H_2_O_2_ leading to a programmed cell death around the point infection that suppresses pathogen growth (Jones and Dangl, 2006).

**Figure 2:**
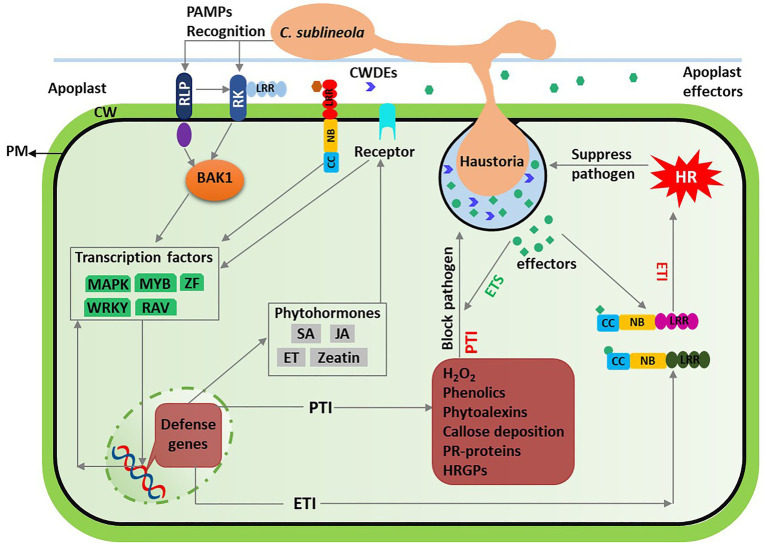
Overview of sorghum-*Colletotrichum sublineola* interactions. Spores of *C. sublineola* land on sorghum tissue, propagate into germ tubes, and form appressoria, which are specialized infection structures. The infection penetrates the host cell wall (CW) and forms feeding structures called haustoria. During the course of infection, pathogenicity molecules, such as cell wall degrading enzymes (CWDEs) and effectors are secreted into the extracellular space. The CWDEs and ubiquitous structural molecules such as chitin are recognized as Pathogen associated molecular patterns (PAMPs) by the host cell receptors, receptor kinases (RK), and receptor like proteins (RLP). These receptors interacts with extracellular leucine rich repeat (LRR) and intracellular BRASSINOSTEROID INSENSITIVE 1-ASSOCIATED KINASE 1 (BAK1) activating the PAMP-triggered immunity (PTI). Host PTI response is characterized by increased accumulation of hydrogen peroxide (H_2_O_2_), phenolics, phytoalexins, hydroxyprolinerich glycoproteins (HRGPs), pathogenesis- related (PR-) proteins, and callose deposition around the point of infection and suppress pathogen growth. In susceptible genotypes, the pathogen secrets effectors that suppress the PTI whereas in resistant genotypes these extracellular and intracellular effectors are recognized by the host nucleotide-binding (NB) LRR (NB-LRR) receptors. This recognition induces the effector-triggered immunity (ETI) primarily recognized by accumulation of H_2_O_2_ resulting in hypersensitive response (HR), which is a form of programmed cell death. The ETI response blocks *C. sublineola* transition to necrotrophic phase and arrests the pathogen growth. Both PTI and ETI responses involve transcriptional factors (green) and phytohormones (gray), involved in signaling transduction, regulation of defense-related genes, and host physiology.

The host NB and LRR domain containing proteins in the resistant genotypes recognize corresponding Avr effectors, described in gene-for-gene interaction. However, the defense response in sorghum involves several other genes as exemplified by a high degree of anthracnose resistance variability among the landraces and the QTLs related to *C. sublineola* resistance (Lebeau et al., 1950; da Costa et al., 2011; Patil et al., 2017; Prom et al., 2018; Ahn et al., 2019). An overview of the interactions involving *C. sublineola* infection and sorghum defense response is presented in Figure 2.

## Major Resistance Genes Conferring Resistance Against *C. sublineola*

Major resistance (*R*-) genes have evolved in plants that directly or indirectly recognize corresponding pathogen secreting small molecules known as effectors. The recognition activates the host resistance response termed ETI. Most of the *R*-genes identified so far in several plants encode proteins containing a central NB and C-terminal LRR domains. Based on their N-terminal domain, NB-LRR encoding genes are grouped into different classes (Mace et al., 2014), but most of the functionally validated *R*-genes in plants so far belong to the coiled-coil domain containing NB-LRR (CC-NB-LRR) type.

Using the sorghum genome sequence (Paterson et al., 2009), Mace et al. (2014) identified more than 346 NB-encoding genes across the 10 chromosomes of the ca 700 Mbp genome. The most recent reannotation that has incorporated additional ca 30 Mbp of sequence has increased the number of genes in the sorghum genome by 24% to 34,211 (McCormick et al., 2018). If these additional sequences are included in the analysis, the number of NB-encoding genes could be even higher. Moreover, recent advancements in plant *R*-gene profiling using single-molecule real-time sequencing of resistance genes (SMRT-RenSeq; Jupe et al., 2014; Witek et al., 2016; Parra et al., 2019) and improving annotation tools like the nucleotide-binding and leucine-rich-repeat NLR-annotator (Steuernagel et al., 2020) may lead to the identification of more *R*-genes in sorghum. Relative to other gene families, the NB-LRR gene family in sorghum shows higher diversity (Mace et al., 2014). This may suggest that a key component of a defense response against fast-evolving pathogens, the NB-LRR genes, is under selection pressure. Interestingly, about 80% of the nucleotide binding site (NBS)-encoding genes are located within the QTL regions of the sorghum genome controlling fungal pathogen resistance (Mace et al., 2014) suggesting that several of these genes could be related to anthracnose resistance in sorghum.

Several studies identified anthracnose resistance QTLs harboring NB-LRR genes in sorghum (Upadhyaya et al., 2013; Felderhoff et al., 2016; Patil et al., 2017; Cuevas et al., 2018, 2019). A sorghum LRR encoding gene associated with anthracnose resistance was found in a locus located on chromosome 6 (Mohan et al., 2010). Using 14,739 SNP markers, Upadhyaya et al. (2013) mapped eight loci linked to anthracnose resistance in sorghum and identified two NB-coding genes on chromosome 10. Genes encoding NB-LRR proteins were among disease resistance genes found in an anthracnose resistance-related QTL located on chromosome 5 (Burrell et al., 2015). Felderhoff et al. (2016) identified NB-LRR protein encoding genes among defense-related genes found within anthracnose resistance loci on chromosome 7 and 9. Patil et al. (2017) detected four NB-LRR genes within a QTL region on chromosome 9 conferring resistance against anthracnose. Cuevas et al. (2018, 2019), respectively identified loci containing CC-NBS-LRR genes in the United States sorghum association panel and Ethiopian sorghum accessions at NPGS. Two NBS-LRR encoding genes, Sobic.008G166400 and Sobic.008G166550, were identified in the QTL region at distal end of chromosome 8 segregating with *C. sublineola* resistance in a sorghum crossing population (Xu, 2019). The known NB-LRR sequences associated with anthracnose resistance in sorghum are summarized in Table 1. Identifying markers closely linked to the QTL regions and developing specific primers for these NB-LRR gene sequences are an important step for efficient identification of landrace genotypes for sorghum breeding. Resistant genotypes with other desirable agronomic traits such as seed yield can be directly deployed for production, or used as a source of resistance trait for improving elite materials using marker-assisted conventional breeding as well as transgenic methods. The resistance trait in the sources genotype can be boosted using genome-editing methods. Therefore, functional validation of the *R*-genes would lead to efficient utilization of the genetic resources for anthracnose resistance improvement.

**Table 1:** The list of quantitative trait loci (QTL) and NB–LRR genes identified in sorghum genotypes responding to anthracnose infection.

QTL/Gene	R-genes (NB-LRR)	Gene identification	Chromosome	Chromosomal position (Mbp)
QTL/Cs1A^1^	NB-LRR	Sb09g027470	SBI-09	4.9–5.05
QTL/Cs2A^1^	CC-NB-LRR	Sb09g004240	SBI-09	56.5–56.6
QTL/Cs1B^1^	CC-NB-LRR	Sb09g027520	SBI-09	4.9–5.1
QTL/Cs2B^1^	CC-NB-LRR	Sb09g004210	SBI-09	56.5–56.6
QTL^1^	CC-NB	Sb09g004215	SBI-09	4.9–5.05
QTL^1^	NB-LRR	Sb09g004220	SBI-09	4.9–5.05
QTL^1^	CC-LRR	Sb09g004230	SBI-09	4.9–5.05
QTL^2^	LRR	Sobic.005G182400	SBI-05	60–72
QTL^2^			SBI-01	60–72
QTL^2^	CC-NB-LRR	Sobic.009G013300	SBI-09	−1.9
	LRR	Sobic.009G012900	SBI-09	−1.9
QTL^3^	NB-LRR	Sobic.007G085400	SBI-07	0–55
QTL^3^			SBI-09	0.5–3.5
QTL^4^			SBI-04	0–11.4
QTL^4^			SBI-06	0–6.3
QTL^4^			SBI-06	39–40.9
QTL^4^			SBI-06	45.2–49
QTL^5^	CC-NB-LRR	Sobic.005G167500	SBI-05	63.68–65.66
	CC-NB-LRR	Sobic.005G167600	SBI-05	63.68–65.66
	CC-NB-LRR	Sobic.005G183000	SBI-05	63.68–65.66
	CC-NB-LRR	Sobic.005G183300	SBI-05	63.68–65.66
QTL^5^	NB_ARC	Sobic.009G013000	SBI-09	57.42–58.36
	NB_ARC	Sobic.009G013100	SBI-09	57.42–58.36
	NB_ARC	Sobic.009G013300	SBI-09	57.42–58.36
Cg1 locus^6^			SBI-05	
QTL^7^	NB-ARC	Sb10g021850	SBI-10	48–48.65
	NB-ARC	Sb10g021860	SBI-10	48–48.65
SbLRR2^8^	LRR	Sb05g018800	SBI-05	55.03–55.04
QTL^9^	NBS-LRR	Sb05g026470	SBI-05	53.80–62.15
	NBS-LRR	Sb05g026480	SBI-05	53.80–62.15

NB, nucleotide binding.

1 Biruma et al. (2012).

2 Cuevas et al. (2018).

3 Felderhoff et al. (2016).

4 Mohan et al. (2010).

5 Patil et al. (2017).

6 Perumal et al. (2009).

7 Upadhyaya et al. (2013).

8 Zhu et al. (2015).

9 Burrell et al. (2015).

A study by Biruma et al. (2012) showed that silencing of two NB-LRR encoding genes, the *Cs1A* and *Cs2A* identified using cDNA-amplified fragment length polymorphism (AFLP) transcript profiling of resistant and susceptible sorghum genotypes, promote the anthracnose infection (Biruma et al., 2012). Moreover, the *SbLRR2*, a gene encoding a simple extracellular LRR protein, showed stronger expression in anthracnose resistant sorghum genotypes compared to susceptible genotypes, suggesting its role in the resistance against anthracnose (Zhu et al., 2015). The NB-LRR genes from common bean (Wu et al., 2017), tea (Shi et al., 2016), strawberry (Liab et al., 2013), and *Arabidopsis* (Birker et al., 2009) were shown to confer resistance against anthracnose in these crops. While these studies showed the role of NB-LRR genes in sorghum anthracnose resistance, their functional validation should confirm if these genes would maintain their function when transferred into a susceptible genotype. Several other sorghum NB-LRR genes need to be isolated, cloned, and transferred into susceptible genotypes and tested if they confer resistance against diverse strains of the pathogen. However, NB-LRR works in gene-for-gene interaction, recognizing cognate effector protein secreted by a strain of the pathogen (van der Biezen and Jones, 1998; Jones and Dangl, 2006). This indicates several NB-LRR and other defense-related genes are required for efficient plant resistance. Hence, isolation and functional characterization of several NBS-LRR gene sequences, localized with the QTLs conferring anthracnose resistance, would increase the *R*-gene resource availability for enhanced anthracnose resistance.

Several sorghum NB-LRR genes were also identified conferring resistance against multiple pathogens. For instance, the sorghum *SbLRR2* that is involved in anthracnose resistance conferred resistance against necrotrophic pathogens *Botrytis cinerea* and *Alternaria brassicicola* in transgenic *Arabidopsis thaliana* (Zhu et al., 2015). This may imply that the sorghum NB-LRR genes that confer resistance to fungal pathogens such as the *Setosphaeria turcica* (Martin et al., 2011) and *Periconia circinata* (Nagy and Bennetzen, 2008) could also confer resistance against anthracnose in sorghum. NB-LRR encoding genes cloned from sorghum and introduced to susceptible rice lines provide resistance to blast disease caused by *Magnaporthe oryzae* (Yang et al., 2013). The sorghum genome also contains homologous genes conferring resistance to common leaf rust (*Rp1*; NB-LRR) in maize (Chavan et al., 2015) and stripe rust resistance (*Yr10*; CC-NB-LRR) in wheat (Liu et al., 2014). Cloning of these genes from anthracnose resistant sorghum genotypes, introducing them to susceptible genotypes followed by their evaluation for anthracnose resistance is vitally important in order to consider their use in sorghum breeding. Such investigation could identify if any of the NB-LRR genes are involved in resistance against multiple pathogens posing threat in sorghum production. However, the plant host resistance does not rely only on the NB-LRR genes to thwart pathogen attack. Hence, the identification of other gene families involved in the plant defense response against the pathogen is equally relevant to facilitate breeding efforts.

## Signaling Cascades and Defense Response Against Anthracnose

Plant defense response involves recognition of the pathogen, signaling transduction, and the response resulting from differential expression of several genes, proteins, and metabolites. The plant defense response commences when host receptors recognize the pathogen PAMPs and effectors. Due to the complexity of the defense response, several defense-related genes are involved in defense against pathogens. Although it does not fully demonstrate their function in anthracnose resistance, several genes, such as F-box domain, peroxidases, and Glucuronosyl transferases, chitinases, germinlike proteins, polyphenol oxidases, peroxidases, ABC-transporters, defensins, and related hypersensitive response have been identified within the loci containing NB-LRR genes (Upadhyaya et al., 2013; Felderhoff et al., 2016; Cuevas et al., 2018). Using suppression subtractive hybridization (SSH), Li et al. (2013) identified several genes involved in signal transduction, secondary metabolism, protein synthesis, and degradation activated in response to *C. sublineola* infection of sorghum genotypes.

### Sorghum Receptors Recognizing *C. sublineola*

Pattern-recognition receptors, such as receptor kinases (RKs) or receptor-like proteins (RLPs), are surface-localized receptors perceiving the pathogen PAMPs and trigger host defense response PTI (Zipfel, 2014). The ETI relies on *R*-receptors, encoded by the NB-LRR genes, which recognizes the pathogen secreted virulence effectors directly or indirectly (Zipfel, 2014). Interestingly, two genes annotated as cysteine-rich RLKs were identified in the NB-LRR genes containing anthracnose resistance associated QTL region on chromosome 5 in sorghum (Patil et al., 2017). RLPs interact with LRR, and LRR-RLP complex requires the BRI1-ASSOCIATED KINASE-1 (*BAK1*) also known as *SERK1* involved in downstream signaling (Liebrand et al., 2014). Due to its broad role in several pathosystems, *BAK1* is considered central regulator of plant innate immunity (Zipfel, 2014). Yazawa et al. (2013) showed that *BAK1* is involved in resistance against a necrotrophic fungal pathogen *Bipolaris sorghicola* causing target leaf spot in sorghum. Although not experimentally validated so far, these results indicate a potential role of the RLKs-LRRs-BAK1 complex in sorghum resistance against anthracnose. Following the pathogen recognition and activation of plant defense, transcription factors regulate expression of the genes involved in downstream signaling and response against the pathogen.

### Transcription Factors Regulating Sorghum Response Against Anthracnose

The host plant defense signaling involves transcription factors that control the expression level of target genes and regulate cellular processes conditioning plant response to stresses (Pandey and Somssich, 2009). Three well-known family of transcription factors, the MYB, WRKY, and MAPK are involved in plant defense against various pathogens (Pandey and Somssich, 2009; Ambawat et al., 2013; Meng and Zhang, 2013). The sorghum genome contains 94 putative WRKY transcription factors (Baillo et al., 2020) as well as 128 MYB and 83 MAPK gene ontologies, found using keyword search in Phytozyme v12.1 (Accessed on December 14, 2020). Some transcription factors are involved in sorghum resistance to anthracnose. A yellow seed1 (*y1*) encoding MYB transcription factor (Chopra et al., 2002; Boddu et al., 2005; Ibraheem et al., 2010) and a putative MYB domain protein 48 (*MYB48*; Patil et al., 2017) regulate the accumulation of phytoalexins and flavonoids in response to anthracnose infection in sorghum. Both antimicrobial compounds accumulate at the site of anthracnose infection (Lo et al., 1999; Basavaraju et al., 2009; Dao et al., 2011). In their recent study, Tugizimana et al. (2018) showed that phenylpropanoid and flavonoid pathways are central hub of the metabolism producing anti-fungal compounds in response to anthracnose infection in sorghum. Li et al. (2013) identified two MAPK transcription factors related genes in anthracnose infected sorghum seedlings. Downregulation of a zinc finger-like transcription factor (Sb03g041170) compromised *C. sublineola* resistance in sorghum (Biruma et al., 2012). A RAV transcription factor (Sb01g049150 in locus 3) involved in *R*-gene resistance mediated pathway (Upadhyaya et al., 2013). Hence, functionally diverse transcriptional factors are involved in the sorghum defense response against anthracnose infection, which is characterized by changes in phytohormone levels (Tugizimana et al., 2018).

### Role of Phytohormones in Anthracnose Resistance

Phytohormones, such as salicylic acid (SA), jasmonic acid (JA), and ethylene (ET), play crucial role in signaling pathways related to plant defense response against biotic and abiotic stresses (Verma et al., 2016). Accumulation of phytohormones, in response to pathogen infection, is perceived by receptor proteins and subsequently initiates intracellular signal transduction and interacts with the transcription factors (Li et al., 2020). A recent study by Tugizimana et al. (2018) revealed quantitative changes of JA, SA conjugates, and abscisic acid (ABA) in anthracnose-infected sorghum. Sorghum genotypes with enhanced levels of amino acids (tyrosine, tryptophan), JA and SA conjugates, and zeatin were more resistant to *C. sublineola* (Tugizimana et al., 2018). Higher expression of chalcone synthase in *C. sublineola* incoluated sorghum (Ahn et al., 2019) could be associated with the accumulation of flavonoid and isoflavonoid phytoalexins and is involved in the salicylic acid mediated defense pathway, as reviewed in Dao et al. (2011). Likewise, JA (but not SA and ET) induced expression of the resistance gene *SbLRR2*, suggesting that it is involved in JA-mediated sorghum defense against the pathogen (Zhu et al., 2015). It is possible that the other resistance genes could be involved in SA and ET mediated defense response against the pathogen. Induction of ABA-responsive genes only in anthracnose inoculated resistant sorghum cultivars (Li et al., 2013) indicates role of the phytohormone in the resistance against the pathogen. However, the interactions among these phytohormones (resulting in synergy or antagonism) have not been discussed in relation to anthracnose resistance in sorghum. Plant response involving the recognition, signaling transduction, transcriptional regulation, phytohormone accumulation, and production of antimicrobial compounds is fine-tuned to the virulence of the infecting pathogen strain. This warrants knowledge about biology of the pathogen populations, pathogenicity, and virulence of its strains. This knowledge is crucial to understand the plant-defense responses and design optimized breeding programs for improving sorghum anthracnose resistance.

## *Colletotrichum sublineola* Causing Anthracnose In Sorghum

*Colletotrichum sublineola*, causal agent of the devastating anthracnose disease in sorghum, is a hemibiotrophic fungal pathogen (Crouch and Beirn, 2009; Tesso et al., 2012). Previously, *C. sublineola* was known as *Colletotrichum graminicola*, but rDNA sequences, restriction fragment length polymorphism (RFLP) of mitochondrial DNA, mating analysis, and appressorial morphology revealed that these two species are distinctly different (Vaillancouri and Hanau, 1992; Sherriff et al., 1995; Wharton and Julian, 1996). *C. sublineola* reproduces asexually but pairing of isolates induces teleomorph stage of sexual reproduction indicating that the pathogen is heterothallic (Vaillancouri and Hanau, 1992). The presence of parasexual processes contributing to high genetic diversity in *C. sublineola* populations was also reported (Chala et al., 2011). This pathogen primarily overwinters as mycelium, acervuli, and sclerotia, alternatively, in the soil, seeds, and decomposing crop residues (Casela and Frederiksen, 1993; Crouch and Beirn, 2009). This indicates that the pathogen is well-adapted to seasonal changes and can easily transmit inoculum to new plants in the subsequent growing season. During favorable conditions, asexual conidia are formed, disseminated through water splash, attached to the plant tissue, and cause a disease (Crouch and Beirn, 2009).

Understanding the life history traits of *C. sublineola* is fundamental for efficient identification of resistance sources and eventual introgression of resistance genes into elite cultivars. The knowledge about the pathogen’s biology and lifecycle is also crucial for designing enhanced disease management strategies. An overview of the pathogen’s biology and lifecycle including its morphology, overwintering and primary inoculum sources, plant-to-plant dissemination mechanisms, and reproductive structures, infection strategy was previously published (Crouch and Beirn, 2009). Elucidating genetic diversity and pathogenicity of *C. sublineola* populations, characteristics related to pathogen’s survival and ability to cause a disease, and its evolutionary potential across major sorghum growing regions is paramount for developing durable resistance in sorghum, as presented in the following sections.

### High-Diversity and Pathogenicity of *C. sublineola* Populations

Amplified fragment length polymorphism analysis revealed the presence of diverse *C. sublineola* isolates within a local field in Ethiopia (Chala et al., 2011; Chala, 2013). Likewise, 13 pathotypes were identified among 87 isolates of the pathogen collected from research stations and production fields in Arkansas, using differential reaction in sorghum genotypes (Moore et al., 2008). The results indicate a high diversity of the pathogen even within a single field. On the other hand, Rosewich et al. (1998) found not much variation within 411 *C. sublneola* isolates sampled from 1991 to 1993 from a sorghum disease nursery in Georgia, United States. As these authors noted, the presence of one predominant haplotype leads to low diversity of the pathogen population in the area. Variability of the pathogen is a significant challenge because it enables the pathogen to adapt to the deployed resistance quickly. Conducive environment, mutation (particularly in the effector proteins), and genetic recombination due to mating, epidemiology, and co-evolution with alternative hosts such as wild relatives as well as resistance deployment can increase pathogen aggressiveness or evolve new virulent strains and cause significant damage. Thus, the diversity of the pathogen within one field warrants breeding cultivars with a broad resistance that can cope with diverse and fast-evolving pathotypes. In addition, introduced resistance should be optimal for diverse sorghum growing areas with contrasting environmental conditions. Unfortunately, research showed that the anthracnose population structure is different across geographically distinct regions and varies across crop growing seasons. Using AFLP analysis, a high level of genetic variation among 102 isolates and genetic differentiation between isolates from different sites in Ethiopia were reported (Chala et al., 2011). More than 230 *C. sublineola* isolates collected from Texas, Arkansas, Georgia, and Puerto Rico in United States, characterized using AFLP markers exhibited a high genetic diversity and differential virulence profiles (Prom et al., 2012b). A RAPD based study revealed a high genetic variation among 37 *C. sublineola* isolates, which were clustered according to their geographic origin in Brazil (Valerio et al., 2005). The virulence characterization of these isolates using differential sorghum genotypes led to the identification of 22 races (Valerio et al., 2005), indicating high diversity of the pathogen in genetic make-up and pathogenicity. Based on the resistance reactions of 15 sorghum inbred lines grown in 14 anthracnose hotspots in Asia and Africa, Thakur et al. (2007) found differences in virulence of the *C. sublineola* populations across the locations and growing seasons. Despite these efforts, given the wide distribution of the pathogen and its diversity, the pathogen populations across sorghum growing regions remains poorly understood. This needs globally coordinated efforts to develop robust genetic markers, set of sorghum differentials for testing pathogenicity as previously suggested (Tesso et al., 2012); and identify and characterize key molecular determinants of the pathogenicity such as the effector proteins (Buiate et al., 2017).

### Deciphering the Role of Effector Proteins in *C. sublineola* Pathogenicity

Plant pathogens secret effector proteins to overcome host defense response and manipulate cell physiology to cause a disease. The whole genome sequences of the pathogen followed by comparative genomics can provide crucial insights into the pathogen evolution and pathogenicity. Such approach also facilitates the development of molecular markers for the identification of different strains of the pathogen. The published *C. sublineola* genome (Baroncelli et al., 2014) contains genes coding for hundreds of secreted small molecules with 180 of them putatively annotated as effectors (Buiate et al., 2017) using the EffectorP prediction tool (Sperschneider et al., 2018). The number of predicted effectors in *C. sublineola* is higher compared to that of its closely related species *C. graminicola* (Buiate et al., 2017), thereby suggesting their more diverse role during the infection process. Similar to the work done in *C. graminicola* effectors in maize (Gong et al., 2020), bioinformatics analysis, isolation, and characterization of the *C. sublineola* effectors, would play a crucial role in elucidating the role of these effectors in determining the virulence of the pathogen and infection process in sorghum. Moreover, the cloning of *C. sublineola* effectors would facilitate future resistance screening of sorghum landraces and wild relatives using effectromics, a method for quick identification of major *R*-genes as shown in potato wild relatives (Domazakis et al., 2017). Characterizing the *C. sublineola* effectors would help efficiently determine the races of the pathogen and follow virulence pattern of *C. sublineola* populations across geographical regions.

## Towards Omics-Aided Anthracnose Resistance Improvement in Sorghum

The sorghum-anthracnose interaction is a complex process, which activates defense response characterized by the change in the expression of several genes (Li et al., 2013), proteins, and metabolites in the host plant (Tugizimana et al., 2018, 2019) and the pathogen. Advances in genomics, transcriptomics, proteomics, and metabolomics studies can help elucidate the cellular processes during the host-pathogen interactions. Following the sequencing of the sorghum reference genome in 2009 (Paterson et al., 2009), several sorghum genotypes have been re-sequenced to differentiate and characterize their agronomic traits (Zheng et al., 2011; Evans et al., 2013) and understand the complex domestication history of the crop (Mace et al., 2013). Likewise, Baroncelli et al. (2014) reported the draft genome sequence of *C. sublineola*. These sorghum and *C. sublineola* genetic and genomic resources are important to understand their biology, identify and understand the genes underlying variation leading to resistance and pathogenicity, and hence facilitate genetic improvement of the crop (Buiate et al., 2017; Boyles et al., 2019). For example, the genome sequence data of both organisms were used for the identification of NB-LRR and other defense-related genes (Biruma et al., 2012; Mace et al., 2014) as well as understanding host-specificity and putative pathogenicity genes in *C. sublineola* (Buiate et al., 2017). Re-sequencing of several other genotypes and strains, and continuous improvement of genome annotation would facilitate the use of functional genomics to understand the pathosystem and enhance sorghum resistance against the pathogen.

**Figure 3:**
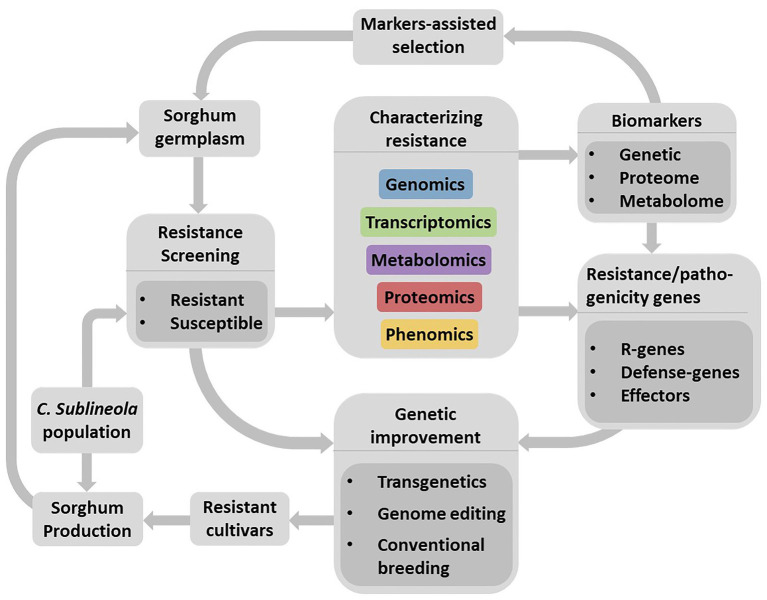
Schematic flow representing a strategy for the development of anthracnose resistant sorghum cultivars through the identification of resistance sources, development and application of biomarkers as well as understanding the pathosystems. Large-scale resistance screening using diverse *C. sublineola* strains would lead to the identification of resistant and susceptible genotypes. Integrated omics is an efficient approach to identify and characterize compatible (susceptibility) and incompatible (resistance) reaction of sorghum genotypes as well as to identify markers related to plant defense and pathogenicity factors. Availability of genomic information on sorghum and *C. sublineola* would enable efficient application of integrated omics approach to unravel their interactions. Validated markers for their association with the target trait can facilitate the identification of resistant genotypes in a diverse sorghum germplasm while defense related genes, particularly major *R*-genes are useful for genetic improvement of sorghum cultivars. However, the ever-evolving pathogen populations continue to pose a challenge to resistant cultivars deployed for production. Hence, it is important to add newly identified resistant cultivars to existing resistance gene pool and frequently re-evaluate their resistance against new *C. sublineola* strains to ensure durability of the resistance.

Existing genome sequence databases for both organisms make it possible to apply integrated omics approaches to understand their interactions, identify resistance sources, and facilitate the development of sorghum cultivars resistant to anthracnose through the application of advanced plant breeding methods (Figure 3). Not all genes contained in the genome but those transcriptionally induced, at particular time during compatible and incompatible sorghum-anthracnose interactions, lead to alterations in myriad of defense-response or pathogenicity related proteins and metabolites. Large-scale transcriptomics, proteomics, and metabolomics studies can capture these changes in the host and pathogen. Increasingly cheaper sequencing technologies and advancing instrumentation, data processing tools, and bioinformatics software make omics studies possible methods in deciphering sorghum-anthracnose interactions. However, it is only recently that transcriptomics (Wang et al., 2020) and metabolomics (Tugizimana et al., 2018) approaches are applied in sorghum–*C. sublineola* interactions. Since several pathogens secrete effectors into the apoplast, considered the frontier of the interaction, investigating the host and pathogen proteins in this compartment would provide insights into early stages of the interaction process.

## Conclusion and Future Perspectives

The sorghum–anthracnose interaction is economically important pathosystem. However, not much is known about the molecular aspects of the interaction processes between these organisms despite its significance in facilitating the improvement of the crop’s resistance against the pathogen. The resistance of diverse sorghum landraces against anthracnose has been evaluated, and useful resistance sources for breeding were identified in several studies. Anthracnose resistance QTL regions are hotspots for potentially functional NB-LRR genes that, after functional validation, can be transferred, individually or by pyramiding multiple resistance genes, into elite sorghum materials. Moreover, the anthracnose defense-related genes identified so far can serve as biomarkers for quick and efficient identification of resistance sources in the diverse sorghum gene pool. The role of receptors, transcription factors, and phytohormones during compatible and incompatible interactions remains to be elucidated. Sorghum breeding efforts should consider spatio-temporal distributions of the diverse *C. sublineola* populations in order to develop resilient cultivars with durable resistance and a wide adaptation. Existing genomic resources of both sorghum and *C. sublineola* is crucial to elucidate the interaction process using omics studies. Hence, the utilization of these resources has to be ramped up for their potential role in facilitating the identification of key components of host resistance and pathogen virulence, tracking dynamics of pathogen populations across seasons and geographical area, and accordingly contributing to accelerated and efficient sorghum breeding.

## Author Contributions

KA conceptualized and drafted the manuscript, with the assistance of MG. KA, MG, RO, and AC reviewed and edited the manuscript. KA prepared the figures and table. All authors contributed to the article and approved the submitted version.

### Conflict of Interest

The authors declare that the research was conducted in the absence of any commercial or financial relationships that could be construed as a potential conflict of interest.

## References

[ref1] Acharya B. O’Quinn T. N. Everman W. Mehl H. L. (2019). Effectiveness of fungicides and their application timing for the management of sorghum foliar anthracnose in the mid-Atlantic United States. Plant Dis. 103, 2804–2811. 10.1094/PDIS-10-18-1867-RE, PMID: 31524095

[ref2] Afolayan G. Deshpande S. P. Aladele S. E. Kolawole A. O. Angarawai I. Nwosu D. J.. (2019). Genetic diversity assessment of sorghum (*Sorghum bicolor* (L.) Moench) accessions using single nucleotide polymorphism markers. Plant Genet. Resour. 17, 412–420. 10.1017/S1479262119000212

[ref3] Ahn E. Prom L. K. Odvody G. Magill C. (2019). Defense responses against the sorghum anthracnose pathogen in leaf blade and midrib tissue of johnsongrass and sorghum. Physiol. Mol. Plant Pathol. 106, 81–86. 10.1016/j.pmpp.2018.12.008

[ref4] Ambawat S. Sharma P. Yadav N. R. Yadav R. C. (2013). MYB transcription factor genes as regulators for plant responses: an overview. Physiol. Mol. Biol. Plants 19, 307–321. 10.1007/s12298-013-0179-1, PMID: 24431500 PMC3715649

[ref5] Ananda G. K. S. Myrans H. Norton S. L. Gleadow R. Furtado A. Henry R. J. (2020). Wild sorghum as a promising resource for crop improvement. Front. Plant Sci. 11:1108. 10.3389/fpls.2020.01108, PMID: 32765575 PMC7380247

[ref6] Baillo E. H. Hanif M. S. Guo Y. H. Zhang Z. B. Xu P. Algam S. A. (2020). Genome-wide identification of WRKY transcription factor family members in sorghum (*Sorghum bicolor* (L.) moench). PLoS One 15:e0236651. 10.1371/journal.pone.0236651, PMID: 32804948 PMC7430707

[ref7] Baroncelli R. Sanz-Martin J. M. Rech G. E. Sukno S. A. Thon M. R. (2014). Draft genome sequence of *Colletotrichum sublineola*, a destructive pathogen of cultivated sorghum. Genome Announc. 2, e00540–e00554. 10.1128/genomeA.00540-14, PMID: 24926053 PMC4056296

[ref8] Basavaraju P. Shetty N. P. Shetty H. S. de Neergaard E. Jorgensen H. J. (2009). Infection biology and defence responses in sorghum against *Colletotrichum sublineolum*. J. Appl. Microbiol. 107, 404–415. 10.1111/j.1365-2672.2009.04234.x, PMID: 19302494

[ref9] Birker D. Heidrich K. Takahara H. Narusaka M. Deslandes L. Narusaka Y.. (2009). A locus conferring resistance to *Colletotrichum higginsianum* is shared by four geographically distinct Arabidopsis accessions. Plant J. 60, 602–613. 10.1111/j.1365-313X.2009.03984.x, PMID: 19686535

[ref10] Biruma M. Martin T. Fridborg I. Okori P. Dixelius C. (2012). Two loci in sorghum with NB-LRR encoding genes confer resistance to *Colletotrichum sublineolum*. Theor. Appl. Genet. 124, 1005–1015. 10.1007/s00122-011-1764-8, PMID: 22143275

[ref11] Boddu J. Svabek C. Ibraheem F. Jones A. D. Chopra S. (2005). Characterization of a deletion allele of a sorghum Myb gene, yellow seed1 showing loss of 3-deoxyflavonoids. Plant Sci. 169, 542–552. 10.1016/j.plantsci.2005.05.007

[ref12] Boora K. S. Frederiksen R. Magill C. (1998). DNA-based markers for a recessive gene conferring anthracnose resistance in sorghum. Crop Sci. 38, 1708–1709. 10.2135/cropsci1998.0011183X003800060048x

[ref13] Boyles R. E. Brenton Z. W. Kresovich S. (2019). Genetic and genomic resources of sorghum to connect genotype with phenotype in contrasting environments. Plant J. 97, 19–39. 10.1111/tpj.14113, PMID: 30260043

[ref14] Buiate E. A. S. Souza E. A. Vaillancourt L. Resende I. Klink U. P. (2010). Evaluation of resistance in sorghum genotypes to the causal agent of anthracnose. Crop Breed. Appl. Biotechnol. 10, 166–172. 10.12702/1984-7033.v10n02a10

[ref15] Buiate E. A. S. Xavier K. V. Moore N. Torres M. F. Farman M. L. Schardl C. L.. (2017). A comparative genomic analysis of putative pathogenicity genes in the host-specific sibling species *Colletotrichum graminicola* and *Colletotrichum sublineola*. BMC Genomics 18:67. 10.1186/s12864-016-3457-9, PMID: 28073340 PMC5225507

[ref16] Burrell A. M. Sharma A. Patil N. Y. Collins S. D. Anderson W. F. Rooney W. L.. (2015). Sequencing of an anthracnose-resistant sorghum genotype and mapping of a major QTL reveal strong candidate genes for anthracnose resistance. Crop Sci. 55, 790–799. 10.2135/cropsci2014.06.0430

[ref17] Casela C. R. Frederiksen R. A. (1993). Survival of *Colletotrichum graminicola Sclerotia* in sorghum stalk residues. Plant Dis. 77, 825–827. 10.1094/Pd-77-0825

[ref18] Chala A. (2013). Genetic diversity of *Colletotrichum sublineolum* isolates from a single field in southern Ethiopia and evidence for the existence of mat2 genotypes in different parts of the country. Ethiop. J. Health Sci. 36, 9–16.

[ref19] Chala A. Tronsmo A. M. Brurberg M. B. (2011). Genetic differentiation and gene flow in *Colletotrichum sublineolum* in Ethiopia, the Centre of origin and diversity of sorghum, as revealed by AFLP analysis. Plant Pathol. 60, 474–482. 10.1111/j.1365-3059.2010.02389.x

[ref20] Chavan S. Gray J. Smith S. M. (2015). Diversity and evolution of Rp1 rust resistance genes in four maize lines. Theor. Appl. Genet. 128, 985–998. 10.1007/s00122-015-2484-2, PMID: 25805314

[ref21] Chopra S. Gevens A. Svabek C. Wood K. V. Peterson T. Nicholson R. L. (2002). Excision of the Candystripe1 transposon from a hyper-mutable Y1-cs allele shows that the sorghumY1 gene controls the biosynthesis of both 3-deoxyanthocyanidin phytoalexins and phlobaphene pigments. Physiol. Mol. Plant Pathol. 60, 321–330. 10.1016/S0885-5765(02)90411-X

[ref22] Crouch J. A. Beirn L. A. (2009). Anthracnose of cereals and grasses. Fungal Divers. 39, 19–44.

[ref23] Crouch J. A. Tomaso-Peterson M. (2012). Anthracnose disease of centipedegrass turf caused by *Colletotrichum eremochloae*, a new fungal species closely related to *Colletotrichum sublineola*. Mycologia 104, 1085–1096. 10.3852/11-317, PMID: 22492402

[ref24] Cuevas H. E. Prom L. K. (2020). Evaluation of genetic diversity, agronomic traits, and anthracnose resistance in the NPGS Sudan Sorghum core collection. BMC Genomics 21:88. 10.1186/s12864-020-6489-0, PMID: 31992189 PMC6988227

[ref25] Cuevas H. E. Prom L. K. Cooper E. A. Knoll J. E. Ni X. (2018). Genome-wide association mapping of anthracnose (*Colletotrichum sublineolum*) resistance in the U.S. sorghum association panel. Plant Genome 11, 1–13. 10.3835/plantgenome2017.11.0099 PMC12962439 30025025

[ref26] Cuevas H. E. Prom L. K. Cruet-Burgos C. M. (2019). Genome-wide association mapping of anthracnose (*Colletotrichum sublineolum*) resistance in NPGS Ethiopian sorghum germplasm. G3 9, 2879–2885. 10.1534/g3.119.400350, PMID: 31289022 PMC6723129

[ref27] Cuevas H. E. Prom L. K. Erpelding J. E. Brotons V. Lübberstedt T. (2014). Assessments of genetic diversity and anthracnose disease response among Zimbabwe sorghum germplasm. Plant Breed. 133, 234–242. 10.1111/pbr.12133

[ref28] Cuevas H. E. Prom L. K. Isakeit T. Radwan G. (2016). Assessment of sorghum germplasm from Burkina Faso and South Africa to identify new sources of resistance to grain mold and anthracnose. Crop Prot. 79, 43–50. 10.1016/j.cropro.2015.10.007

[ref29] Cuevas H. E. Rosa-Valentin G. Hayes C. M. Rooney W. L. Hoffmann L. (2017). Genomic characterization of a core set of the USDA-NPGS Ethiopian sorghum germplasm collection: implications for germplasm conservation, evaluation, and utilization in crop improvement. BMC Genomics 18:108. 10.1186/s12864-016-3475-7, PMID: 28125967 PMC5270221

[ref30] da Costa R. V. Zambolim L. Cota L. V. da Silva D. D. Rodrigues J. A. S. Tardin F. D.. (2011). Genetic control of sorghum resistance to leaf anthracnose. Plant Pathol. 60, 1162–1168. 10.1111/j.1365-3059.2011.02469.x

[ref31] Dao T. T. H. Linthorst H. J. M. Verpoorte R. (2011). Chalcone synthase and its functions in plant resistance. Phytochem. Rev. 10, 397–412. 10.1007/s11101-011-9211-7, PMID: 21909286 PMC3148432

[ref32] Das I. K. Rajendrakumar P. (2016). “Disease resistance in Sorghum” in Biotic stress resistance in millets. eds. Das I. K. Padmaja P. G. (Academic Press), 23–67.

[ref33] Domazakis E. Lin X. Aguilera-Galvez C. Wouters D. Bijsterbosch G. Wolters P. J.. (2017). Effectoromics-based identification of cell surface receptors in potato. Methods Mol. Biol. 1578, 337–353. 10.1007/978-1-4939-6859-6_29, PMID: 28220439

[ref34] Erpelding J. (2008). Sorghum germplasm resistance to anthracnose. Am. J. Plant Sci. Biotechnol. 2, 42–46.

[ref35] Evans J. McCormick R. F. Morishige D. Olson S. N. Weers B. Hilley J.. (2013). Extensive variation in the density and distribution of DNA polymorphism in sorghum genomes. PLoS One 8:e79192. 10.1371/journal.pone.0079192, PMID: 24265758 PMC3827139

[ref36] FAOSTAT (2020). Food and Agriculture Organization of the United Nations. Available at: http://www.fao.org/faostat/en/ (Accessed November 20, 2020).

[ref37] Felderhoff T. J. McIntyre L. M. Saballos A. Vermerris W. (2016). Using genotyping by sequencing to map two novel anthracnose resistance loci in *Sorghum bicolor*. G3 6, 1935–1946. 10.1534/g3.116.030510, PMID: 27194807 PMC4938647

[ref38] Fesel P. H. Zuccaro A. (2016). Beta-glucan: crucial component of the fungal cell wall and elusive MAMP in plants. Fungal Genet. Biol. 90, 53–60. 10.1016/j.fgb.2015.12.004, PMID: 26688467

[ref39] Girma G. Nida H. Seyoum A. Mekonen M. Nega A. Lule D.. (2019). A large-scale genome-wide association analyses of Ethiopian sorghum landrace collection reveal loci associated with important traits. Front. Plant Sci. 10:691. 10.3389/fpls.2019.00691, PMID: 31191590 PMC6549537

[ref40] Gong A. D. Jing Z. Y. Zhang K. Tan Q. Q. Wang G. L. Liu W. D. (2020). Bioinformatic analysis and functional characterization of the CFEM proteins in maize anthracnose fungus *Colletotrichum graminicola*. J. Integr. Agric. 19, 541–550. 10.1016/S2095-3119(19)62675-4

[ref41] Ibraheem F. Gaffoor I. Chopra S. (2010). Flavonoid phytoalexin-dependent resistance to anthracnose leaf blight requires a functional yellow seed1 in *Sorghum bicolor*. Genetics 184, 915–926. 10.1534/genetics.109.111831, PMID: 20083611 PMC2865927

[ref42] Jones J. D. Dangl J. L. (2006). The plant immune system. Nature 444, 323–329. 10.1038/nature05286, PMID: 17108957

[ref43] Jupe F. Chen X. W. Verweij W. Witek K. Jones J. D. G. Hein I. (2014). Genomic DNA library preparation for resistance gene enrichment and sequencing (RenSeq) in plants. Methods Mol. Biol. 1127, 291–303. 10.1007/978-1-62703-986-4_22, PMID: 24643569

[ref44] Kage U. Kumar A. Dhokane D. Karre S. Kushalappa A. C. (2016). Functional molecular markers for crop improvement. Crit. Rev. Biotechnol. 36, 917–930. 10.3109/07388551.2015.1062743, PMID: 26171816

[ref45] Lebeau F. J. Coleman O. H. Agon J. (1950). The inheritance of resistance in sorghum to leaf anthracnose. CABI Direct 42, 33–34.

[ref46] Li T. Gonzalez N. Inze D. Dubois M. (2020). Emerging connections between small RNAs and phytohormones. Trends Plant Sci. 25, 912–929. 10.1016/j.tplants.2020.04.004, PMID: 32381482

[ref47] Li L. Zhu F. Y. Liu H. J. Chu A. Lo C. (2013). Isolation and expression analysis of defense-related genes in sorghum-*Colletotrichum sublineolum* interaction. Physiol. Mol. Plant Pathol. 84, 123–130. 10.1016/j.pmpp.2013.08.005

[ref48] Liab J. Zhang Q.-Y. Gaob Z.-H. Wang F. Duan K. Ye Z.-H.. (2013). Genome-wide identification and comparative expression analysis of NBS–LRR-encoding genes upon *Colletotrichum gloeosporioides* infection in two ecotypes of *Fragaria vesca*. Gene 527, 215–227. 10.1016/j.gene.2013.06.008, PMID: 23806759

[ref49] Liebrand T. W. H. van den Burg H. A. Joosten M. H. A. J. (2014). Two for all: receptor-associated kinases SOBIR1 and BAK1. Trends Plant Sci. 19, 123–132. 10.1016/j.tplants.2013.10.003, PMID: 24238702

[ref50] Little C. R. Prom L. K. Perumal R. Tesso T. T. Odvody G. N. Magill C. W. (2012). Sorghum pathology and biotechnology—A fungal disease perspective: part I. grain mold, head smut, and ergot. Eur. J. Plant Sci. Biotechnol. 6, 31–44.

[ref51] Liu W. Frick M. Huel R. Nykiforuk C. L. Wang X. M. Gaudet D. A.. (2014). The stripe rust resistance gene Yr10 encodes an evolutionary-conserved and unique CC-NBS-LRR sequence in wheat. Mol. Plant 7, 1740–1755. 10.1093/mp/ssu112, PMID: 25336565

[ref52] Lo S. C. C. De Verdier K. Nicholson R. L. (1999). Accumulation of 3-deoxyanthocyanidin phytoalexins and resistance to *Colletotrichum sublineolum* in sorghum. Physiol. Mol. Plant Pathol. 55, 263–273. 10.1006/pmpp.1999.0231

[ref53] Mace E. S. Tai S. S. Gilding E. K. Li Y. H. Prentis P. J. Bian L. L.. (2013). Whole-genome sequencing reveals untapped genetic potential in Africa’s indigenous cereal crop sorghum. Nat. Commun. 4:2320. 10.1038/ncomms3320, PMID: 23982223 PMC3759062

[ref54] Mace E. Tai S. S. Innes D. Godwin I. Hu W. S. Campbell B.. (2014). The plasticity of NBS resistance genes in sorghum is driven by multiple evolutionary processes. BMC Plant Biol. 14:253. 10.1186/s12870-014-0253-z, PMID: 25928459 PMC4189741

[ref55] Martin T. Biruma M. Fridborg I. Okori P. Dixelius C. (2011). A highly conserved NB-LRR encoding gene cluster effective against *Setosphaeria turcica* in sorghum. BMC Plant Biol. 11:151. 10.1186/1471-2229-11-151, PMID: 22050783 PMC3262770

[ref56] McCormick R. F. Truong S. K. Sreedasyam A. Jenkins J. Shu S. Sims D.. (2018). The *Sorghum bicolor* reference genome: improved assembly, gene annotations, a transcriptome atlas, and signatures of genome organization. Plant J. 93, 338–354. 10.1111/tpj.13781, PMID: 29161754

[ref57] Mehta P. J. Wiltse C. C. Rooney W. L. Collins S. D. Frederiksen R. A. Hess D. E.. (2005). Classification and inheritance of genetic resistance to anthracnose in sorghum. Field Crop Res. 93, 1–9. 10.1016/j.fcr.2004.09.001

[ref58] Meng X. Z. Zhang S. Q. (2013). MAPK cascades in plant disease resistance signaling. Annu. Rev. Phytopathol. 51, 245–266. 10.1146/annurev-phyto-082712-102314, PMID: 23663002

[ref59] Mengistu G. Shimelis H. Laing M. Lule D. (2018). Breeding for anthracnose (*Colletotrichum sublineolum* Henn.) resistance in sorghum: challenges and opportunities. Aust. J. Crop. Sci. 12, 1911–1920. 10.21475/ajcs.18.12.12.p1230

[ref60] Mengistu G. Shimelis H. Laing M. Lule D. (2019). Assessment of sorghum genetic resources of Ethiopia for anthracnose (*Colletotrichum sublineolum* Henn.) resistance and agronomic traits. J. Phytopathol. 167, 667–678. 10.1111/jph.12861

[ref61] Mengistu G. Shimelis H. Laing M. Lule D. Mathew I. (2020). Genetic variability among Ethiopian sorghum landrace accessions for major agro-morphological traits and anthracnose resistance. Euphytica 216:113. 10.1007/s10681-020-02650-6

[ref62] Mohan S. M. Madhusudhana R. Mathur K. Chakravarthi D. V. N. Rathore S. Reddy R. N.. (2010). Identification of quantitative trait loci associated with resistance to foliar diseases in sorghum [*Sorghum bicolor* (L.) Moench]. Euphytica 176, 199–211. 10.1007/s10681-010-0224-x

[ref63] Moore J. W. Ditmore M. TeBeest D. O. (2008). Pathotypes of *Colletotrichum sublineolum* in Arkansas. Plant Dis. 92, 1415–1420. 10.1094/PDIS-92-10-1415, PMID: 30769577

[ref64] Motlhaodi T. Geleta M. Chite S. Fatih M. Ortiz R. Bryngelsson T. (2017). Genetic diversity in sorghum [*Sorghum bicolor* (L.) Moench] germplasm from southern Africa as revealed by microsatellite markers and agro-morphological traits. Genet. Resour. Crop. Evol. 64, 599–610. 10.1007/s10722-016-0388-x

[ref65] Nagy E. D. Bennetzen J. L. (2008). Pathogen corruption and site-directed recombination at a plant disease resistance gene cluster. Genome Res. 18, 1918–1923. 10.1101/gr.078766.108, PMID: 18719093 PMC2593579

[ref66] Ordonio R. Ito Y. Morinaka Y. Sazuka T. Matsuoka M. (2016). Molecular breeding of *Sorghum bicolor*, A novel energy crop. Int. Rev. Cell Mol. Biol. 321, 221–257. 10.1016/bs.ircmb.2015.09.001, PMID: 26811289

[ref67] Panday S. Sindhu A. Boora K. (2002). RAPD based DNA markers linked to anthracnose disease resistance in *Sorghum bicolor* (L.) Moench. Indian J. Exp. Biol. 40, 206–211. PMID: 12622185

[ref68] Pandey S. P. Somssich I. E. (2009). The role of WRKY transcription factors in plant immunity. Plant Physiol. 150, 1648–1655. 10.1104/pp.109.138990, PMID: 19420325 PMC2719123

[ref69] Parra L. B. Truco M. J. Fletcher K. Michelmore R. W. (2019). Identification of candidate genes for resistance to lettuce downy mildew using Renseq k-mer association studies. Mol. Plant-Microbe Interact. 32:7.

[ref70] Paterson A. H. Bowers J. E. Bruggmann R. Dubchak I. Grimwood J. Gundlach H.. (2009). The *Sorghum bicolor* genome and the diversification of grasses. Nature 457, 551–556. 10.1038/nature07723, PMID: 19189423

[ref71] Patil N. Y. Klein R. R. Williams C. L. Collins S. D. Knoll J. E. Burrell A. M.. (2017). Quantitative trait loci associated with anthracnose resistance in sorghum. Crop Sci. 57, 877–890. 10.2135/cropsci2016.09.0793

[ref72] Perumal R. Menz M. A. Mehta P. J. Katile S. Gutierrez-Rojas L. A. Klein R. R.. (2009). Molecular mapping of Cg1, a gene for resistance to anthracnose (*Colletotrichum sublineolum*) in sorghum. Euphytica 165, 597–606. 10.1007/s10681-008-9791-5

[ref73] Prom L. Cuevas H. Ramasamy P. Thomas I. Clint M. (2018). Inheritance of resistance of three sorghum lines to pathotypes of *Colletotrichum sublineola*, causal agent of anthracnose. Plant Pathol. J. 17, 75–79. 10.3923/ppj.2018.75.79

[ref74] Prom L. Erpelding J. Perumal R. Isakeit T. Cuevas H. (2012a). Response of sorghum accessions from four African countries against *Colletotrichum sublineolum*, causal agent of sorghum anthracnose. Am. J. Plant Sci. 3, 125–129. 10.4236/ajps.2012.31014

[ref75] Prom L. K. Perumal R. Erattaimuthu S. R. Little C. R. No E. G. Erpelding J. E.. (2012b). Genetic diversity and pathotype determination of *Colletotrichum sublineolum* isolates causing anthracnose in sorghum. Eur. J. Plant Pathol. 133, 671–685. 10.1007/s10658-012-9946-z

[ref76] Rosewich U. L. Pettway R. E. McDonald B. A. Duncan R. R. Frederiksen R. A. (1998). Genetic structure and temporal dynamics of a *Colletotrichum graminicola* population in a sorghum disease nursery. Phytopathology 88, 1087–1093. 10.1094/Phyto.1998.88.10.1087, PMID: 18944821

[ref77] Sherriff C. Whelan M. J. Arnold G. M. Bailey J. A. (1995). rDNA sequence analysis confirms the distinction between *Calletotrichum graminicola* and *C. sublineolum*. Mycol. Res. 99, 475–178. 10.1016/S0953-7562(09)80649-7

[ref78] Shi H. Wang L. Wang Y. Cao H. Hao X. Zeng J.. (2016). Transcriptome analysis of an anthracnose-resistant tea plant cultivar reveals genes associated with resistance to *Colletotrichum camelliae*. PLoS One 11:e0148535. 10.1371/journal.pone.0148535, PMID: 26849553 PMC4743920

[ref79] Singh M. Chaudhary K. Singal H. R. Magill C. W. Boora K. S. (2006). Identification and characterization of RAPD and SCAR markers linked to anthracnose resistance gene in sorghum [*Sorghum bicolor* (L.) Moench]. Euphytica 149, 179–187. 10.1007/s10681-005-9065-4

[ref80] Sperschneider J. Dodds P. N. Gardiner D. M. Singh K. B. Taylor J. M. (2018). Improved prediction of fungal effector proteins from secretomes with EffectorP 2.0. Mol. Plant Pathol. 19, 2094–2110. 10.1111/mpp.12682, PMID: 29569316 PMC6638006

[ref81] Steuernagel B. Witek K. Krattinger S. G. Ramirez-Gonzalez R. H. Schoonbeek H. J. Yu G. T.. (2020). The NLR-annotator tool enables annotation of the intracellular immune receptor repertoire. Plant Physiol. 183, 468–482. 10.1104/pp.19.01273, PMID: 32184345 PMC7271791

[ref82] Tesso T. Perumal R. Little C. R. Adeyanju A. Ghada L. R. Prom L.. (2012). Sorghum pathology and biotechnology—a fungal disease perspective: part II. Anthracnose, stalk rot, and downy mildew. Eur. J. Plant Sci. Biotechnol. 6, 31–34.

[ref83] Thakur R. Rao V. Wu S. Subbara K. Mathur K. Tailor H.. (2007). Genetic resistance to foliar anthracnose in sorghum and pathogenic variability in *Colletotrichum graminicola*. Indian Phytopathol. 60, 13–23.

[ref84] Tugizimana F. Djami-Tchatchou A. T. Steenkamp P. A. Piater L. A. Dubery I. A. (2018). Metabolomic analysis of defense-related reprogramming in *Sorghum bicolor* in response to *Colletotrichum sublineolum* infection reveals a functional metabolic web of phenylpropanoid and flavonoid pathways. Front. Plant Sci. 9:1840. 10.3389/fpls.2018.01840, PMID: 30662445 PMC6328496

[ref85] Tugizimana F. Steenkamp P. A. Piater L. A. Labuschagne N. Dubery I. A. (2019). Unravelling the metabolic reconfiguration of the post-challenge primed state in *Sorghum bicolor* responding to *Colletotrichum sublineolum* infection. Meta 9:194. 10.3390/metabo9100194, PMID: 31547091 PMC6835684

[ref86] Upadhyaya H. D. Narsimha Reddy K. Vetriventhan M. Irshad Ahmed M. Murali Krishna G. Thimma Reddy M.. (2017). Sorghum germplasm from west and Central Africa maintained in the ICRISAT genebank: status, gaps, and diversity. Crop J. 5, 518–532. 10.1016/j.cj.2017.07.002

[ref87] Upadhyaya H. D. Vetriventhan M. Deshpande S. (2016). “Sorghum germplasm resources characterization and trait mapping” in Sorghum Genome. eds. Rakshit S. Wang Y. H. (Cham: Springer), 77–94.

[ref88] Upadhyaya H. D. Wang Y. H. Sharma R. Sharma S. (2013). Identification of genetic markers linked to anthracnose resistance in sorghum using association analysis. Theor. Appl. Genet. 126, 1649–1657. 10.1007/s00122-013-2081-1, PMID: 23463493

[ref89] Vaillancouri L. Hanau R. (1992). Genetic and morphological cotnparisons of *Glomerella* (*Colletotrichum*) isolates from maize and from sorghum. Exp. Mycol. 16, 219–222. 10.1016/0147-5975(92)90030-U

[ref90] Valerio H. M. Resende M. A. Weikert-Oliveira R. C. B. Casela C. R. (2005). Virulence and molecular diversity in *Colletotrichum graminicola* from Brazil. Mycopathologia 159, 449–459. 10.1007/s11046-005-0373-y, PMID: 15883732

[ref91] van der Biezen E. A. Jones J. D. G. (1998). Plant disease-resistance proteins and the gene-for-gene concept. Trends Biochem. Sci. 23, 454–456. 10.1016/S0968-0004(98)01311-5, PMID: 9868361

[ref92] Verma V. Ravindran P. Kumar P. P. (2016). Plant hormone-mediated regulation of stress responses. BMC Plant Biol. 16:86. 10.1186/s12870-016-0771-y, PMID: 27079791 PMC4831116

[ref93] Wang L. Chen M. Zhu F. Fan T. Zhang J. Lo C. (2020). Alternative splicing is a *Sorghum bicolor* defense response to fungal infection. Planta 251:14. 10.1007/s00425-019-03309-w, PMID: 31776670

[ref94] Wang M. L. Dean R. Erpelding J. Pederson G. (2006). Molecular genetic evaluation of sorghum germplasm differing in response to fungal diseases: rust (*Puccinia purpurea*) and anthracnose (*Collectotrichum graminicola*). Euphytica 148, 319–330. 10.1007/s10681-005-9040-0

[ref95] Wharton P. Julian A. (1996). A cytological study of compatible and incompatible interactions between *Sorghum bicolor* and *Colletotrichum sublineolum*. New Phytol. 134, 25–34. 10.1111/j.1469-8137.1996.tb01143.x

[ref96] Wharton P. Julian A. O’Connell R. (2001). Ultrastructure of the infection of *Sorghum bicolor* by *Colletotrichum sublineolum*. Phytopathology 91, 149–158. 10.1094/PHYTO.2001.91.2.149, PMID: 18944388

[ref97] Witek K. Jupe F. Witek A. I. Baker D. Clark M. D. Jones J. D. G. (2016). Accelerated cloning of a potato late blight-resistance gene using RenSeq and SMRT sequencing. Nat. Biotechnol. 34, 656–660. 10.1038/nbt.3540, PMID: 27111721

[ref98] Wu J. Zhu J. F. Wang L. F. Wang S. M. (2017). Genome-wide association study identifies NBS-LRR-encoding genes related with anthracnose and common bacterial blight in the common bean. Front. Plant Sci. 8:1398. 10.3389/fpls.2017.01398, PMID: 28848595 PMC5552710

[ref99] Xu X. (2019). Identification and mapping of anthracnose resistance genes in sorghum [*Sorghum bicolor* (L.) moench]. PhD thesis. Faculty of Purdue University.

[ref100] Xu J. Qin P. Jiang Y. Hu L. Liu K. Xu X. (2020). Evaluation of sorghum germplasm resistance to anthracnose by *Colletotrichum sublineolum* in China. Crop Prot. 134:105173. 10.1016/j.cropro.2020.105173

[ref101] Yang S. H. Li J. Zhang X. H. Zhang Q. J. Huang J. Chen J. Q.. (2013). Rapidly evolving R genes in diverse grass species confer resistance to rice blast disease. Proc. Natl. Acad. Sci. U. S. A. 110, 18572–18577. 10.1073/pnas.1318211110, PMID: 24145399 PMC3831948

[ref102] Yazawa T. Kawahigashi H. Matsumoto T. Mizuno H. (2013). Simultaneous transcriptome analysis of Sorghum and *Bipolaris sorghicola* by using RNA-seq in combination with de novo transcriptome assembly. PLoS One 8:e62460. 10.1371/journal.pone.0062460, PMID: 23638091 PMC3640049

[ref103] Zheng L. Y. Guo X. S. He B. Sun L. J. Peng Y. Dong S. S.. (2011). Genome-wide patterns of genetic variation in sweet and grain sorghum (*Sorghum bicolor*). Genome Biol. 12:R114. 10.1186/gb-2011-12-11-r114, PMID: 22104744 PMC3334600

[ref104] Zhu F. Y. Li L. Zhang J. H. Lo C. (2015). Transgenic expression of a sorghum gene (SbLRR2) encoding a simple extracellular leucine-rich protein enhances resistance against necrotrophic pathogens in *Arabidopsis*. Physiol. Mol. Plant Pathol. 91, 31–37. 10.1016/j.pmpp.2015.05.004

[ref105] Zipfel C. (2014). Plant pattern-recognition receptors. Trends Immunol. 35, 345–351. 10.1016/j.it.2014.05.004, PMID: 24946686

